# No evidence of reactivity in accelerometry‐based measurement of physical activity and sleep

**DOI:** 10.1111/bjhp.70047

**Published:** 2025-12-30

**Authors:** Jan A. Häusser, Janina U. Janurek, Sascha Etgen, Andreas Mojzisch

**Affiliations:** ^1^ Justus‐Liebig‐University Giessen Giessen Germany; ^2^ University of Applied Sciences, Bonn‐Rhein‐Sieg Sankt Augustin Germany; ^3^ University of Hildesheim Hildesheim Germany

**Keywords:** accelerometry, Hawthorne effect, physical activity, reactivity, sleep

## Abstract

**Objective:**

Accelerometry provides more objective estimates of physical activity and sleep parameters than self‐report data. However, the use of accelerometry may suffer from reactivity to measurement, that is, changes in behaviour under study due to the awareness of being observed.

**Design:**

In two field‐experimental studies, we examined reactivity to measurement in physical activity and sleep parameters estimated with accelerometry. We used within‐subjects designs and manipulated if participants were made aware of the measurement intention or if it was concealed.

**Methods:**

During a 2‐week study, participants received a red accelerometer for one of the weeks and were informed that their physical activity would be monitored in this week. In the other week, participants received a black accelerometer and were informed that it would monitor sleep. In fact, both the red and the black accelerometers monitored both physical activity and sleep.

**Results:**

We consistently found no evidence for reactivity to measurement for any indicator of physical activity (moderate to vigorous physical activity, steps) or sleep (duration, efficiency).

**Conclusion:**

Accelerometry can provide unbiased measures of indicators of physical activity and sleep and is therefore a valuable addition to the common use of self‐report data.

In health psychology, a reliable and unbiased measurement of health‐related behaviour is crucial for theory development and practical applications. However, the awareness that one's behaviour is subject to observation can alter the execution or presentation of this behaviour (König et al., 2025). If you observe something, it tends to change—a phenomenon that has been dubbed reactivity to measurement (e.g., French & Sutton, 2010). Obviously, reactivity to measurement is a major problem in research on human behaviour: Due to self‐serving biases, selective attention and social desirability, participants who are aware that their behaviour is being measured in a study tend to be motivated to alter the behaviour under study in a more favourable direction—thereby seriously jeopardizing the validity of the measurement (Donaldson & Grant‐Vallone, 2002; see French & Sutton, 2010 for a review).

In the behavioural sciences, the discussion of this measurement problem dates back to the yet famous Hawthorne studies (see Jung & Lee, 2015, and Haslam & Steffens, 2023 for recent discussions). Almost a century ago, between 1924 and 1932, Elton Mayo and colleagues conducted a series of field experiments involving employees who made electrical relays at the Hawthorne Works in Cicero, Illinois (Mayo, 1933, 1945; Roethlisberger & Dickson, 1939). The field experiments aimed at increasing productivity in the electric plant by, for example, changing lighting conditions or implementing break schedules. The studies claimed to have shown that the researchers' attention towards the workers tended to improve the workers' productivity―independently of the specific type of interventions (and even in the control conditions). While these findings are highly disputed in the scientific community (e.g., Levitt & List, 2011), in later discussions of the Hawthorne studies, the findings have been interpreted in terms of reactivity to measurement, sometimes also dubbed as the ‘Hawthorne Effect’ (Landsberger, 1958).

## PHYSICAL ACTIVITY AND SLEEP

Two types of broader health‐related behaviours have attracted much attention in recent years: physical activity and sleep. Physical activity has been found to have tremendous effects on diverse health and well‐being outcomes, such as all‐cause mortality across large‐scale samples and prospective studies (e.g., Feng et al., 2023; Löllgen et al., 2009; Martinez‐Gomez et al., 2024; Nocon et al., 2008). Meta‐analyses reveal that this relationship is non‐linear (Martinez‐Gomez et al., 2024), differs at different intensity levels, with a strongest impact from no to low or moderate activity (Löllgen et al., 2009), and depends on timing, for example, time of day when physical activity is executed (Feng et al., 2023). This renders a fine‐grained and reliable measurement of physical activity crucial in the study of physical activity as a health behavior. Similarly, suboptimal sleep has a major impact on health and well‐being, including all‐cause mortality (e.g., Cappuccio et al., 2010). For some indicators, such as sleep duration, the relationships with health are non‐linear (Shen et al., 2016). At the same time, sleep is a multi‐faceted construct (Lee & Lawson, 2021), encompassing different indicators of quantity (e.g., sleep duration, timing) and interruptions (e.g., sleep efficiency, awakenings). Hence, for sleep, a fine‐grained, reliable and multi‐dimensional measure is of high importance.

However, because the substantial impact of physical activity and sleep is so well documented, these health behaviours have a strong normative dimension; this renders these behaviours as prescriptive accompanied by explicit behavioural recommendations, regarding sufficient physical activity (cf. World Health Organization, 2020) and sleep (cf. Watson et al., 2015). Due to this normative nature, reactivity to measurement is likely to occur in examinations of physical activity and sleep as a result of self‐enhancement (Dufner et al., 2019) and self‐presentation motivations (Schlenker, 2012). Hence, self‐report measures of health behaviour are likely to be biased because, in addition to reactivity to measurement, that is, an actual change in behaviour, systematic over‐ or under‐reporting as well as memory biases also blur the measurement of true behaviour (Baranowski, 1985; Donaldson & Grant‐Vallone, 2002).

A promising solution to this problem might lie in the application of technical devices to ‘objectively’ monitor the health behaviour under study. In particular, a very common and well‐established non‐self‐report approach to monitor physical activity and sleep is tri‐axial accelerometry, also sometimes referred to as actigraphy (Littner et al., 2003; Reilly et al., 2008). With this technique, small devices are attached to the bodies of the participants (typically either at the hip or at the wrist), containing high‐sensitivity sensors that capture acceleration of the body in a three‐dimensional space. These acceleration patterns can be translated back to specific types of (non‐)movement using algorithms.

While the use of accelerometry should solve the problems of motivated over‐ and under‐reporting as well as memory biases, it does not solve the problem that participants might *actually* alter their health‐related behaviour. In particular, the introduction of a new device to monitor one's own behaviour can per se have an effect on the behaviour to be monitored, even in the absence of social desirability and researcher expectation effects. König et al. (2025) argue that an explicit awareness of being observed by a researcher is not a necessary condition for reactivity of measurement to occur, but that the mere novelty of being monitored by a technical device naturally increases the salience and relevance of the observed behaviour, leading to changes in this behaviour. Thus, familiarity with device‐based measurement of behaviour can mitigate reactivity to measurement (Arigo & König, 2024; König et al., 2022). In the following, we will discuss how accelerometry‐based measurement of physical activity and sleep may produce reactivity to measurement.

## HOW BEING MONITORED MAY INFLUENCE PHYSICAL ACTIVITY

Even though most people are aware of the beneficial effects of physical activity, they still fail to fulfil recommendations for minimum physical activity, as, for example, issued by the World Health Organization (2020). This discrepancy creates a situation where reactivity to measurement is highly likely to occur as a consequence of social desirability and self‐serving motives, in addition to changes in behaviour due to the introduction of a new technical monitoring device (cf. König et al., 2022). In studies of physical activity that employ technical devices such as accelerometry, participants are fully aware of their study participation and the measurement of physical activity as target behaviour is highly salient. At the same time, they are aware of desired outcomes (sufficient physical activity) and might therefore be tempted to increase their otherwise low physical activity to reach the desired outcome (for self‐approval as well as for reasons of impression management). Based on these considerations, we put forward the following hypothesis:

Hypothesis 1 Individuals show reactivity to accelerometry‐based physical activity measurement. When individuals are aware that their physical activity is monitored, they will show increased physical activity as compared to when they are not aware that their physical activity is monitored.

Previous research provides tentative empirical evidence for reactivity to measurement in technology‐based examinations of physical activity. A recent meta‐analysis by König et al. (2022) synthesized studies on reactivity to measurement when using technical devices to monitor physical activity (pedometers and actigraphy accelerometers) and found a mean effect of Cohen's *d* = .27, hinting at a small but significant effect.

However, a closer inspection of the studies included in the meta‐analysis provides a more nuanced picture and reveals some methodological shortcomings of previous research. In particular, the confidence intervals of 13 of the 18 effect sizes presented in the meta‐analysis included zero (König et al., 2022). Hence, reactivity to measurement was found in only a minority of about 28% of the tests. Strikingly, the average effect of *d* = .27 was largely driven by two studies that used pedometers and reported medium to large reactivity to measurement effects (*d* = .84 in Clemes et al., 2008, and *d* = .65 in Clemes & Parker, 2009). Moreover, the studies in the meta‐analyses used a frequentist statistical approach, which can only be used to reject the null hypothesis but cannot be used to prove (or accept) it (i.e., the absence of evidence is not evidence of absence). This limitation applies to the null findings of the studies included in the meta‐analysis. In contrast, in our research, we use a probabilistic Bayesian approach to test our research hypothesis against the null hypothesis.

Beyond this obvious lack of robustness in published reactivity to measurement effects, there is a fundamental practical problem that refers to the adequate control condition used in this line of research: In order to judge if reactivity to measurement has occurred, a comparison condition is required in which physical activity is unaffected by measurement—but how does one measure physical activity that is *unaffected* by measurement? To solve this paradoxical situation, some studies used manipulations aiming at increasing reactivity to measurement in order to test its occurrence. For example, studies used additional devices to induce reactivity to measurement (e.g., Foley et al., 2011; Ho et al., 2013) or used feedback from unsealed devices (e.g., Clemes et al., 2008; Foote et al., 2017). However, these approaches are not well suited to inform about reactivity to measurement per se.

Another line of research used within‐subject designs with longer study periods (7–84 days), examining trajectories in physical activity after the onset of measurement (e.g., Craig et al., 2010; Klenk et al., 2019; Tinlin et al., 2018; Ullrich et al., 2021). In these studies, a decline in steps or increase in sedentary behaviour over time is interpreted as an indicator of initial reactivity to measurement because participants are assumed to return to pre‐assessment levels of physical activity over the course of measurement. The majority of these studies found no decline in physical activity over the period of study (cf. König et al., 2022), which could be interpreted as either (a) no reactivity to measurement or (b) no decline in reactivity to measurement over time. Moreover, it is unclear if the strong assumption of a complete return to the baseline is justified.

The most reasonable approach is to conceal the measurement intention (physical activity) of the accelerometer in one condition and to compare it to a condition where the participants are informed about the measurement intention. Vanhelst et al. (2017) used this approach and presented the accelerometer as a device that allegedly measured body posture (see Clemes & Parker, 2009 for a similar procedure with pedometers). However, this study used a between‐subjects design (which is problematic due to interindividual variability in physical activity) and was somewhat underpowered (*n* = 40) so that the insignificant difference between conditions is difficult to interpret (without Bayesian statistics).

## HOW BEING MONITORED MAY INFLUENCE SLEEP

Although it has been suggested that sleep monitoring itself could be considered an ‘intervention’ causing changes in sleep behaviour (e.g., Kölling & Hof zum Berge, 2020), predictions for the specific form of reactivity to measurement of sleep are less straightforward, as compared to physical activity, for at least three reasons. First, in contrast to physical activity, parameters such as sleep duration or awakening during the night lack volitional control to some degree. Of course, similar to physical activity, participants should be aware that ‘good sleep’ is beneficial for health and psychological functioning. However, potential ways to reach this objective are restricted. That is, even if participants are motivated to alter their sleep behaviour, they cannot simply decide to sleep better or longer (as they could for exercising more intensely or more often).

Second, being monitored might even produce negative effects for sleep: Being equipped with a device to track sleep should increase the focus on sleep (or sleep problems), which may cause irritations and negatively affect sleep (Harvey, 2002). Hence, it is even possible that reactivity to measurement does not occur in the form of a motivational driver to reach desired behavioural outcomes but instead may increase irritation.

Third, when compared to physical activity, sleep should be subject to social desirability and self‐serving attempts to a lesser degree, because sleep does not follow a simple the‐more‐the‐merrier function (as might be assumed for physical activity by lay persons). Hence, participants should have weaker assumptions about desired outcomes, and therefore, they should be less motivated to alter their sleeping behaviour in one direction or the other.

All things considered, with respect to sleep, we refrain from formulating a directed hypothesis, but put forward an open research question:

*Research Question*: Do individuals show reactivity to accelerometry‐based sleep measurement? Thus, will the awareness that their sleep is monitored affect individuals' length and efficiency of sleep?

In the light of the lack of clear‐cut predictions regarding reactivity to measurement in assessments of sleep, it does not come as a surprise that empirical evidence is very sparse. To the best of our knowledge, only two studies have examined potential reactivity in accelerometry‐based sleep measurement: In a between‐subjects experimental study with university students, Kölling and Hof zum Berge (2020) found that accelerometry‐measured sleep was affected by additional portable polysomnography measurement, with increased sleep efficiency in the nights with portable polysomnography measurement as compared to the accelerometry‐only nights. The authors interpreted this finding as indicating that participants were more aware of going to sleep and were therefore tempted to avoid distracting behaviours. Moreover, it was found that, when compared to a non‐monitored control condition, accelerometry (together with sleep logs and portable polysomnography measurement) did not alter participants' attitudes and beliefs about sleep. This, however, does not inform about reactivity to measurement in the accelerometry‐measured sleep per se. A second study (Cizza et al., 2014) used the screening and randomization phases of a sleep intervention study to examine reactivity to measurement. They found a slight increase in sleep duration from screening to randomization (prior to any intervention, but after the study protocol and study group had been revealed). However, similar to the observational within‐subject studies for physical activity (see above), this design is not well‐suited to inform about causal effects of accelerometry measurement on sleep behaviour.

## PRESENT RESEARCH

In order to capture physical activity and sleep, accelerometers can be attached to different body locations, specifically the wrist or the hip (Tryon, 2011; see Quante et al., 2015 for a detailed discussion). Previous research has found that hip‐based accelerometry provides more reliable estimations of physical behaviour compared to hip‐based accelerometry (Trost et al., 2005; Tryon, 2011; see Tudor‐Locke et al., 2015, for a direct comparison). At the same time, however, hip‐based accelerometry tends to overestimate sleep duration because inactive non‐sleep activities (e.g., lying on the couch) may be classified as sleeping (Hjorth et al., 2012). Besides differential overestimation, the estimates from both body locations correspond highly with each other (for physical activity, see Kamada et al., 2016; Mandigout et al., 2019; Scott et al., 2017; for sleep see Full et al., 2018). To account for the risk of differential overestimation associated with body location, we decided to run a series of two studies: one using wrist‐worn accelerometers (Study 1) and one using hip‐worn accelerometers (Study 2).

Moreover, we deem it a crucial challenge of reactivity to measurement studies to provide a control condition in which physical activity and sleep are monitored with accelerometry without participants' awareness. To overcome this problem, both studies used manipulation of the alleged measurement intention in within‐person designs: In 1 week, participants received a red accelerometer and were informed that it would continuously monitor their physical activity during this complete week. In the other week, participants received a black accelerometer and were informed that it would monitor their sleep during this complete week. In fact, the red and the black accelerometers were identical (with the exception of colour) and they monitored both physical activity and sleep. This allowed us to monitor one type of health behaviour (e.g., physical activity) while participants firmly believed that we would only monitor the other type of health behaviour (e.g., sleep). By doing so, we were able to establish a baseline in which participants by definition should show no reactivity for one type of health behaviour because they believed that the other type of health behaviour would be measured exclusively. The order of the communicated measurement intention was randomly varied between participants.

We report how we determined our sample size, all data exclusions, all manipulations, and all measures in the study. The raw data and analyses for both studies can be found at: https://osf.io/tkx3z/?view_only=90aa40dd54bc44aebf7a6828e4010ca1.

## STUDY 1

In Study 1, accelerometers were attached to the wrist, which may result in an overestimation of physical activity, but provides more reliable measures of sleep, as compared to hip‐based measurement.

### Method

#### Participants and design

One hundred and eight undergraduate students took part in a 2‐week field experiment with continuous accelerometry assessment of physical activity (moderate to vigorous physical activity and step counts) and sleep (duration and efficiency). The study included a within‐subjects manipulation of the alleged measurement intention (physical activity vs. sleep). As outlined above, in 1 week, participants received a red accelerometer and were informed that it would continuously monitor their physical activity during this complete week. In the other week, participants received a black accelerometer and were informed that it would monitor sleep during this complete week. The order of the communicated measurement intention was randomly varied between participants and participants were told at the beginning of the study about the different measurements in the two upcoming weeks. To maintain participants' awareness of the alleged measurement intention throughout the week, participants answered short daily questionnaires that asked questions about either their physical activity (when wearing the red device) or their sleep (when wearing the black device) on each day. To not introduce measurement awareness in the control condition, no physical activity questions were asked in the ‘sleep week’ (black accelerometer) and no sleep‐related questions were asked in the ‘physical activity week’ (red accelerometer). Thus, these self‐report data could not be used for between‐condition comparisons. All participants gave their informed consent. As compensation for their participation, participants received €15 or course credit. All participants matched our pre‐defined inclusion criteria for participation (no regular physical activity of more than 5 hours per week, no graduate psychology students, no previous participation in accelerometry studies). Fourteen participants had to be excluded from the analysis because of insufficient data sets (less than 50% of physical activity or sleep data).

The final sample comprised 94 undergraduate students (93% female) with an average age of 21.6 years (SD = 4.64). This sample size was sufficient to detect small to medium effects (Faul et al., 2009) with *α* = .05 and a power of .80 in our within‐person design. Precicely, regarding physical activty, the the sample size was sufficient to detect Cohen’s *d* < .25 (one‐tailed test; which is about the effect size reported in the meta‐analysis by König et al., 2022). For sleep the sample size was sufficient to detect *d* < .29 (two‐tailed). The study received ethical approval by the institutional review board of the last author's university.

#### Measures

Physical activity and sleep were assessed using accelerometry (*ActiSleep +* and *wGT3X‐BT* devices, ActiGraph LLC). The small and lightweight accelerometer devices monitor tri‐axial spatial movements (Tryon, 2011). Devices were attached to participants' wrists on the non‐dominant body side in order to avoid overestimation of movement behaviour (Dieu et al., 2017). Participants wore the accelerometers continuously (day and night) for 15 days (with a change of the devices and the alleged measurement intention on day 8). The days that were not monitored completely (the first and the eighth day of each assessment week) were excluded from the analysis of physical activity, leaving six full days of 24 h per assessment week for each participant.

We used *ActiLife 6* (ActiGraph Software Department, 2020) to calculate two indicators of physical activity, that is, step counts and time spent in moderate to vigorous physical activity (MVPA; in minutes), and two indicators of sleep, that is, total sleep duration in minutes and sleep efficiency. Physical activity parameters were averaged per person over all six full days of assessment week 1 and assessment week 2, respectively. Thus, we calculated the average daily physical activity for the two respective weeks. Sleep parameters were averaged per person over all seven nights of assessment week 1 and assessment week 2, respectively. Thus, we calculated the average daily sleep duration and efficiency in the two respective weeks.

##### MVPA

Following established algorithms (Freedson et al., 1998), every 60 s interval with ≥1952 counts (sample rate 30 Hz) was categorized as MVPA.

##### Steps

Total number of steps was obtained from accelerometry. Step counts are based on the vertical axis of the accelerometer data (ActiGraph Software Department, 2012).

##### Total sleep duration

Total sleep duration in minutes was obtained from accelerometry using established algorithms (Cole et al., 1992; cf. ActiGraph Software Department, 2020).

##### Sleep efficiency

Sleep efficiency is the ratio of time in bed to time asleep. Hence, it is defined by the number of sleep minutes divided by the total number of minutes the participant was in bed (ActiGraph Software Department, 2012).

### Results

We conducted paired *t*‐tests (one‐tailed for physical activity, two‐tailed for sleep)^1^ and calculated Bayes factors using *JASP* (version 0.17.2.1 JASP Team, 2023) in order to investigate potential effects of the alleged measurement intention on indicators of physical activity and sleep.

#### MVPA

Participants had an average MVPA of M = 178.83 min (SD = 44.48) in the ‘physical activity’ week (i.e., when the alleged measurement intention was ‘physical activity’) and M = 182.93 min (SD = 50.65) in the ‘sleep’ week. This difference was not significant, *t*(93) = −1.048, *p*_one‐tailed_ = .851. The Bayes factor analysis revealed that this pattern of results was nearly 17 times more likely to be obtained under the H0 as compared to the H1 (BF01_one‐tailed_ = 16.945).

#### Steps

Step counts amounted to M = 11,254.62 (SD = 2104.50) in the ‘physical activity’ week and M = 11,332.87 (SD = 2396.02) in the ‘sleep’ week. This difference was not significant, *t*(93) = −.397, *p*_one‐tailed_ = .654. The Bayes factor analysis revealed that this pattern of results was nearly 12 times more likely to be obtained under the H0 as compared to the H1 (BF01_one‐tailed_ = 11.658).

#### Total sleep duration

Participants had an average sleep duration of M = 429.66 min (SD = 49.59) in the ‘sleep’ week (i.e., when the alleged measurement intention was ‘sleep’) and M = 431.24 min (SD = 54.13) in the ‘physical activity’ week. This difference was not significant, *t*(93) = −.294, *p*_two‐tailed_ = .769. The Bayes factor analysis revealed that this pattern of results was more than eight times more likely to be obtained under the H0 as compared to an undirected alternative hypothesis (BF01_two‐tailed_ = 8.406).

#### Sleep efficiency

Participants' average sleep efficiency was M = 91.29% (SD = 3.18) in the ‘sleep’ week and M = 91.24% (SD = 3.21) in the ‘physical activity’ week. This difference was not significant, *t*(93) = .239, *p*_two‐tailed_ = .812. The Bayes factor analysis revealed that this pattern of results was more than eight times more likely to be obtained under the H0 as compared to an undirected alternative hypothesis (BF01_two‐tailed_ = 8.527).

#### Additional explorative analyses

To further explore our data, we ran additional analyses, in particular, we conducted (a) separate within‐person comparisons for the first day under study (cf. Arigo & König, 2024), (b) between‐person tests (*t*‐tests for independent samples) for the first study week and (c) repeated measure analyses to account for trajectories over the week. Theses analyses can be found in the OSF project. In sum, these more fine‐grained analyses revealed no evidence for reactivity to measurement.

### Discussion

In a field‐experimental study with a manipulation of the alleged measurement intention, we found no evidence for reactivity to measurement in accelerometry‐based indicators of physical activity and sleep. For all indicators, empirical data were at least eight times more plausible under the H0 as compared to under the alternative hypotheses. Thus, when participants believed that their physical activity was monitored, they were no more physically active than when they believed that their sleep was monitored. Similarly, when participants believed that their sleep was monitored, they slept neither longer (or shorter) nor had a higher (or lower) sleep efficiency than when they believed that their physical activity was monitored. This means that accelerometry is likely to provide measures of physical activity and sleep that are unbiased with respect to participants' awareness of being monitored.

However, our inferences should also account for two potential methodological limitations of our study. First, wrist‐based accelerometry tends to overestimate physical activity because the wrist is a rather active part of the body involved in many types of activities that are of low physical effort (e.g., scratching your head). While we have no reason to assume that this overestimation led to differential effects in the experimental conditions, even an unsystematic overestimation might have produced noise in the data, which would have made it unlikely to find an effect. Therefore, the findings should be replicated using a more valid measure of physical activity (i.e., using hip‐based accelerometry).

A second potential limitation of Study 1 is that participants were informed in advance about the complete experimental procedure. Thus, before starting monitoring of the first week, they were already informed about the upcoming measurement intention of the second week. Hence, we cannot exclude that participants, in an anticipatory manner, might have adapted their behaviours (physical activity and sleep) right away (as compared to an unobserved ‘baseline’), thereby reducing potential differences between experimental conditions. For example, if participants had learned at the beginning of the study that their physical activity would be measured in the second week, they might have already increased their physical activity in the first week.

## STUDY 2

The results of Study 1 provide no evidence for reactivity to the measurement of physical activity and sleep using accelerometry. Study 2 aims to replicate Study 1, testing for the robustness of the null findings. In contrast to Study 1, in Study 2, accelerometers were attached to the hip, which may result in an overestimation of sleep, but provides more valid measures of physical activity as compared to wrist‐based measurement (see above). We had no reason to assume that overestimation would influence reactivity (or interact with reactivity) other than producing noise. Moreover, in contrast to Study 1, participants were not informed about the two alleged measurement intentions of the 2 weeks in advance, but only right at the start of each week.

### Method

#### Participants and design

Ninety‐one students took part in a 2‐week field experiment with a similar mixed design as in Study 1. To overcome the shortcomings of Study 1, two major changes were implemented in the design of Study 2. First, participants wore the accelerometer at the hip instead of the wrist, providing a more conservative measure of physical activity. Second, in contrast to Study 1 and in order to prevent anticipatory reactivity, at the beginning of Study 2, participants were not aware that the measurement would change in the second week. Instead, participants were told that they were part of a study focusing exclusively on physical activity *or* sleep (depending on the order of measurement). They were asked to return to the lab after 1 week to recharge the accelerometers. When arriving at the lab, they were told that sufficient data had already been collected, and they were invited to join another study instead focusing on the other type of behaviour (sleep or physical activity, respectively).

All participants gave their informed consent. As compensation for their participation, they received €20 and had the chance to take part in a lottery of six €100 vouchers. All participants matched our pre‐defined inclusion criteria for participation (no psychology or sports science students, no regular swimming). Nine participants had to be excluded from the analysis because of incomplete data sets (less than 50% of physical activity or sleep data). An additional seven participants had to be excluded because they reported sickness during data assessment, which prevented physical activity and may have altered sleep.

The final sample comprised 75 undergraduate students (86.7% female) with an average age of 23.72 years (SD = 4.30). This sample size was sufficient to detect small to medium effects (Faul et al., 2009); Cohen's *d* < .29, one‐tailed for physical activity, and *d* < .32, two‐tailed for sleep, with *α* = .05 and a power of .80 in our within‐person design. The study received ethical approval by the institutional review board of the last author's university.

#### Measures

Similar to Study 1 (see above for details), physical activity (MVPA and steps) and sleep parameters (total sleep duration and sleep efficiency) were assessed using accelerometry (*ActiSleep +* and *wGT3X‐BT* devices, ActiGraph LLC). However, in contrast to Study 1, participants wore the devices on the hip of their non‐dominant body side. Again, all indicators were averaged per person over all six full days (physical activity) or seven nights (sleep) of assessment week 1 and assessment week 2.

### Results

Similar to Study 1, we conducted paired *t*‐tests (one‐tailed for physical activity and two‐tailed for sleep)^2^ and calculated Bayes factors using *JASP* (version 0.17.2.1; JASP Team, 2023) in order to investigate potential effects of the alleged measurement intention on the outcome variables (i.e., MVPA, step counts, sleep duration and sleep efficiency).

#### MVPA

Participants had an average MVPA of M = 40.44 min (SD = 21.23) in the ‘physical activity’ week (i.e., when the alleged measurement intention was ‘physical activity’) and M = 39.23 min (SD = 20.72) in the ‘sleep’ week. This difference was not significant, *t*(74) = .690, *p*_one‐tailed_ = .246. The Bayes factor analysis revealed that the pattern of results was more than four times more likely to be obtained under the H0 as compared to the H1 (BF01_one‐tailed_ = 4.173).

#### Steps

Step counts amounted to M = 7467.55 (SD = 2565.73) in the ‘physical activity’ week and M = 7372.08 (SD = 2831.28) in the ‘sleep’ week. This difference was not significant, *t*(74) = .418, *p*_one‐tailed_ = .339. The Bayes factor analysis revealed that the pattern of results was more than five times more likely to be obtained under the H0 as compared to the H1 (BF01_one‐tailed_ = 5.491).

#### Total sleep duration

Participants had an average sleep duration of M = 637.07 min (SD = 106.39) in the ‘sleep’ week (i.e., when the alleged measurement intention was ‘sleep’) and M = 627.11 min (SD = 108.66) in the ‘physical activity’ week. This difference was not significant, *t*(74) = .891, *p*_two‐tailed_ = .376. The Bayes factor analysis revealed that the pattern of results was more than five times more likely to be obtained under the H0 as compared to an undirected alternative hypothesis (BF01_two‐tailed_ = 5.372).

#### Sleep efficiency

Participants' average sleep efficiency was M = 95.15% (SD = 1.63) in the ‘sleep’ week and M = 95.10% (SD = 1.67) in the ‘physical activity’ week. This difference was not significant, *t*(74) = .287, *p*_two‐tailed_ = .775. The Bayes factor analysis revealed that the pattern of results was more than seven times more likely to be obtained under the H0 as compared to an undirected alternative hypothesis (BF01_two‐tailed_ = 7.561).

#### Additional explorative analyses

Additional analyses with (a) first‐day analyses, (b) between‐person tests for week 1 and (c) repeated measures analyses can be found in the OSF project. In sum, these analyses revealed no evidence for reactivity to measurement.

### Discussion

Replicating the null findings of Study 1, Study 2 found no evidence for reactivity to accelerometry‐based measurement of physical activity and sleep. Importantly, in contrast to Study 1, Study 2 used a stronger design with no in‐advance information for participants regarding the measurement intentions to prevent anticipatory reactivity. The estimates for physical activity indicators in Study 2 were substantially lower compared to the estimates of Study 1, which may point to the expected overestimation in Study 1 due to body location (wrist). Likewise, the estimates for sleep duration were higher in Study 2, as compared to Study 1, pointing to the expected overestimation of sleep duration in Study 2.

## GENERAL DISCUSSION

In two field experiments, we found no evidence for the occurrence of reactivity to measurement in accelerometry‐based measurement of physical activity (MVPA and steps) and sleep (duration and efficiency). Bayes factor analyses revealed that our data were clearly and consistently in favour of the null hypothesis as compared to the alternative hypotheses for all our indicators. Our findings are encouraging because they demonstrate that the application of accelerometry in the field is likely to produce behavioural measures that are unbiased by changes in behaviour induced by the measurement itself. Thereby our findings contribute to the ongoing debate regarding the validity of accelerometry in physical activity research (e.g., Rothney et al., 2008) and sleep research (e.g., Sadeh, 2011).

Although few earlier studies have reported evidence for reactivity of measurement in accelerometry‐based assessment (cf. König et al., 2022), these effects were rather small and highly inconsistent. More importantly, many previous studies used observational within‐subjects designs (e.g., Baumann et al., 2018; Haegele et al., 2020), which are correlational in nature (correlation of physical activity/sleep and time of measurement) and therefore cannot establish causation. Furthermore, these studies have to rely on the theoretical assumption of a ‘return to baseline’ over time. In addition, most previous studies with experimental designs used additional devices (e.g., Foley et al., 2011; Ho et al., 2013), or used feedback or diary entries to induce reactivity to measurement (e.g., Clemes et al., 2008; Foote et al., 2017). Therefore, these cannot inform if reactivity to measurement would occur when the devices are used in the most reasonable way (i.e., covert, no feedback). Our studies therefore provide an important extension to the existing body of literature, by providing a clear‐cut experimental test of reactivity to measurement without any manipulation to produce or increase reactivity to measurement. Using a Bayesian approach, our findings reveal that reactivity is unlikely to occur when accelerometers are employed in the most reasonable way.

When interpreting the null findings in our study, previous evidence speaking for and against reactivity to measurement should be considered (see König et al., 2022 for a meta‐analysis). The null effects in our studies do not imply that the phenomenon of reactivity to measurement does not exist at all. There are several potential reasons for why it may occur under certain conditions while it does not under others (see also below for a discussion of generalizability). One such condition might be the general level of physical activity in the study population. In our studies, the samples showed relatively high degrees of physical activity that were meeting the World Health Organization's recommendations (WHO, 2020). That is, ceiling effects might have played a role if reactivity of measurement was shown particularly by those who experience a deficit in their physical activity (or sleep duration) and were motivated to increase it. In contrast, under certain conditions, the likelihood of reactivity of measurement should be even systematically reduced, for example, for individuals with prior experience with tracking devices. König et al. (2025) found that the introduction of researcher expectations did not increase reactivity to measurement in a population that was already tracking their physical activity with their own tracking devices. Thus, baseline physical activity (and sleep), motivational premises and experience with (self‐)monitoring target behaviour might critically shape the occurrence and form of reactivity to measurement. Understanding such moderators would also substantially contribute to a better theoretical understanding of *why* reactivity to measurement can occur: is it merely due to increased salience of target behaviour, due to fulfilling researcher expectations, due to social desirability or due to self‐enhancement motives? We deem these important questions for future research as well as important considerations for best‐practice applications of accelerometry in research settings.

In general, as compared to self‐report measures, technology‐based measurement of health‐related behaviours might be better suited to examine quantitative indicators, because there is mounting evidence that individuals tend to over‐report potentially healthy and desired behaviours such as physical activity (see Sallis & Saelens, 2000). However, when it comes to qualitative aspects of such behaviours, accelerometry has clear limitations. For example, for the case of sleep, accelerometry does not allow restfulness or dream content to be examined; in the case of physical activity, it can only monitor duration, frequency and intensity of physical activity but cannot distinguish between different types of physical activity (e.g., running around with vs. without chasing a ball), which may have differential health implications (e.g. risk of injury). To gain a full picture of participants' physical activity and sleep, we would recommend combining both measurement approaches with accelerometry focusing on quantitative indicators and self‐reports focusing on qualitative aspects.

## LIMITATIONS

The present research is not without limitations. Our studies can only inform about reactivity on the behavioural level, that is, about whether or not participants altered their behaviour due to the awareness of being observed during a study. This, however, does not inform about potential reactivity on the motivational level. Due to the awareness of being monitored, participants might have developed an intention to alter their behaviour (e.g., plan to walk instead of using the car), but they may have failed to translate this intention into actual behaviour, for example, due to little self‐efficacy (see Maher et al., 2024 for a discussion of measurement reactivity effects on motivational antecedents of behaviour).

Moreover, in our studies, we recruited a student sample. Because reactivity to measurement is a fundamental and context‐independent psychological phenomenon, we have no reason to assume why reactivity to measurement (or the absence of it as revealed in our studies) should be any different in a student sample as compared to the general population or other broader populations of interest (e.g., employees). Besides consisting of students, our samples were rather young (early twenties) and predominantly female, yielding potentially reduced generalizability regarding age and gender. However, Arigo and König (2024) argued that reactivity to measurement is a rather general phenomenon and the likelihood of strong (unspecific demographic) moderators is therefore low. In line with this, the meta‐analysis by König et al. (2022) revealed only very little evidence for gender effects (with only one out of six studies showing reactivity to measurement in girls, but not boys) and no systematic age effects at all. Nonetheless, for some specific populations, such as individuals with sleep disorders, individuals attempting to establish physical activity routines to lose weight, or individuals with no prior experience with tracking devices and self‐monitoring (cf. König et al., 2025), reactivity to accelerometry‐based measurement is more plausible and our studies should be replicated within these populations.

## CONCLUSION

In conclusion, using Bayesian statistics, our research found no evidence that accelerometry produces reactivity effects in the measurement of physical activity and sleep. Therefore, we clearly recommend its application in the field to obtain unbiased and more objective measures of behaviour.

## AUTHOR CONTRIBUTIONS

**Jan A. Häusser:** Conceptualization; writing – original draft; funding acquisition; investigation; formal analysis; supervision. **Janina U. Janurek:** Formal analysis; methodology; writing – review and editing; project administration; data curation. **Sascha Etgen:** Data curation; formal analysis; writing – review and editing. **Andreas Mojzisch:** Conceptualization; methodology; investigation; funding acquisition; writing – review and editing.

## FUNDING INFORMATION

This research was supported by a grant (HA 6455/3‐2) from the German Research Foundation awarded to JAH and AM.

## CONFLICT OF INTEREST STATEMENT

The authors have no conflict of interest.

## ETHICS STATEMENT

The study was conducted in accordance with the Declaration of Helsinki and the guidelines of the German Psychological Society. The study received ethical approval by the institutional review board of the last author's university.

## Supporting information


Data S1.

## Data Availability

The raw data and analyses for both studies included in this paper can be found at: https://osf.io/tkx3z/?view_only=90aa40dd54bc44aebf7a6828e4010ca1.

## Footnotes

^1^
Given our directional hypothesis for physical activity, one‐tailed testing is most adequate. However, as a robustness check, we reran all analyses with two‐tailed testing. This did not substantially alter the findings and interpretations with MVPA: *p*_two‐tailed_ = .297, BF01 = 5.158; steps: *p*_two‐tailed_ = .692, BF01 = 8.122.

^2^
Given our directional hypothesis for physical activity, again one‐tailed testing is most adequate. However, as a robustness check, we reran all analyses with two‐tailed testing. This did not substantially alter the findings and interpretations with MVPA: *p*_two‐tailed_ = .493, BF01 = 6.259; steps: *p*_two‐tailed_ = .677, BF01 = 7.234.
