# Supplementary material for: No evidence of reactivity in accelerometry‐based measurement of physical activity and sleep
Source: Br J Health Psychol. 2025 Dec 30;31(1):e70047. doi: 10.1111/bjhp.70047 (PMC12750485; doi:10.1111/bjhp.70047)
Supplement: Supplementary file 1 — Data S1. [file BJHP-31-0-s001.docx]

**Supplementary Material for the paper:**

**No Evidence of Reactivity in Accelerometry-Based Measurement of Physical Activity and Sleep**

**Exploratory Analysis 1: Measurement Reactivity on the First Day of Measurement**

As an additional analysis, we conducted two-tailed paired t-tests to investigate potential effects of the alleged measurement intention on indicators of physical activity and sleep on the first day of the study week. This approach was motivated by recent findings from Arigo and König (2024), which suggest that measurement reactivity may be particularly pronounced on the initial day of data collection. Notably, this day was excluded from our primary analyses due to incomplete data coverage (for further details, see the Methods section of the manuscript). To account for different starting times of measurement at the first day, we corrected physical activity metrics—moderate-to-vigorous physical activity (MVPA) and step count—for earing time (physical activity relative to remaining time of the day, that is MVPA and steps per minute).

These analyses indicated no evidence of reactivity to measurement for any indicator of physical activity or sleep on the first day of the measurement (see Supplementary Table 1).

| **Supplementary Table 1**  *Measurement Reactivity on the First Day (Paired T-Tests)* | | | | | | | | |
| --- | --- | --- | --- | --- | --- | --- | --- | --- |
| Outcome | Study 1 | | | | Study 2 | | | |
|  | Mean T1  (SD) | Mean T2  (SD) | *t*  (df) | *p* | Mean T1  (SD) | Mean T2  (SD) | *t*  (df) | *p* |
| MVPA | 0.167  (0.08) | 0.169  (.08) | 0.25  (91) | .806 | 0.06  (0.05) | 0.06  (.04) | 0.53  (73) | .596 |
| Step Count | 10.59  (3.58) | 10.73  (3.81) | 0.27  (91) | .787 | 8.46  (4.84) | 8.78  (4.80) | 0.56  (73) | .578 |
| Sleep Duration | 452.37  (96.60) | 457.72  (114.79) | 0.37  (88) | .710 | 675.05  (172.37) | 682.00  (187.03) | 0.33  (72) | .743 |
| Sleep Efficiency | 91.45  (4.49) | 91.32  (4.37) | 0.32  (88) | .748 | 94.88  (3.16) | 94.90  (2.79) | 0.04  (72) | .966 |
| *Note*. MVPA = moderate to vigorous physical activity (relative to the remaining time of the day). *N_Study1_* = 94; *N_Study2_* = 75. | | | | | | | | |
|  | | | | | | | | |

**Supplementary Analysis 2: First-Week Between-Person Comparison**

To rule out spillover effects, that is a contamination of second-week measurement by the effects of the manipulation on the first week, in a between-person approach, we tested the effects of the alleged measurement intention manipulation in the first week. Therefore, we conducted additional between-person analyses for week one (because the order of alleged measurement intention was counterbalanced about half of the participants started with the “physical activity” week and the other half with the “sleep week”). First, we calculated mean values for each outcome in the first study week. Then, we used two-tailed independent t-tests to assess whether physical activity or sleep differed depending on the condition (i.e., measurement intention).

These analyses were performed separately for Study 1 and Study 2. However, because the between-person approach substantially reduces statistical power, additionally, we used a pooled sample combining participants from both studies. Across all samples, no significant differences emerged between conditions for any of the outcome variables (see Supplementary Table 2).

| **Supplementary Table 2**  *Measurement Reactivity with a Focus on Week One (Independent T-Tests)* | | | | | | | | | | | | |
| --- | --- | --- | --- | --- | --- | --- | --- | --- | --- | --- | --- | --- |
| Outcome | Study 1 | | | | Study 2 | | | | Study 1 & 2 (pooled) | | | |
|  | Mean T1  (SD) | Mean T2  (SD) | *t*  (df) | *p* | Mean T1  (SD) | Mean T2  (SD) | *t*  (df) | *p* | Mean T1  (SD) | Mean T2  (SD) | *t*  (df) | *p* |
| MVPA | 188.50  (46.45) | 175.33  (47.12) | 1.36  (92) | .176 | 42.66  (21.62) | 37.83  (19.43) | 1.01  (73) | .314 | 119.45  (81.66) | 112.35  (78.44) | 0.59  (174) | .557 |
| Step Count | 11450  (2211) | 11332  (2162) | 0.26  (92) | .795 | 7724  (3068) | 7057  (2225) | 1.07  (73) | .287 | 9765  (3289) | 9396  (3056) | 0.77  (174) | .442 |
| Sleep Duration | 470.35  (64.66) | 472.40  (55.06) | 0.16  (90) | .870 | 648.89  (115.19) | 667.62  (136.36) | 0.64  (72) | .524 | 553.80  (126.41) | 653.49  (140.31) | 0.48  (171) | .634 |
| Sleep Efficiency | 91.27  (3.48) | 90.76  (2.93) | 0.78  (90) | .439 | 95.27  (1.64) | 95.02  (1.83) | 0.64  (72) | .528 | 93.15  (3.43) | 92.71  (3.23) | 0.88  (171) | .380 |
| *Note*. MVPA = moderate to vigorous physical activity. *N_Study1_* = 94; *N_Study2_* = 75. | | | | | | | | | | | | |

**Supplementary Analysis 3: Trajectories across the Week**

To explore whether the effects of the manipulation varied across the course of the study week, we conducted repeated-measures ANOVAs with *a)* day (6 days) and *b)* condition (measurement intention) as within-person factors. The outcomes included MVPA, step count, sleep time, and sleep efficiency. An interaction between time and condition would indicate that the alleged measurement intention manipulation would result in different trajectories.

Across all outcomes, no significant day × condition interactions were observed, indicating that trajectories over the study week did not differ between conditions (see Supplementary Table 3).

| **Supplementary Table 3**  *Trajectories over the Week Differentiated by Condition* | | | | | | | |
| --- | --- | --- | --- | --- | --- | --- | --- |
| Outcome | Effect | Study 1 | | | Study 2 | | |
|  |  | df | *F* | *p* | df | *F* | *p* |
| MVPA | Condition | 1, 93 | 1.10 | .297 | 1, 74 | 0.48 | .493 |
|  | Day | 5, 465 | 9.12 | <.001 | 5, 370 | 2.26 | <.05 |
|  | Condition x Day | 5, 465 | 0.73 | .602 | 5, 370 | 1.10 | .359 |
| Step Count | Condition | 1, 93 | 0.16 | .692 | 1, 74 | 0.18 | .677 |
|  | Day | 5, 465 | 12.61 | <.001 | 5, 370 | 2.19 | .055 |
|  | Condition x Day | 5, 465 | 0.17 | .973 | 5, 370 | 0.62 | .683 |
| Sleep Efficiency | Condition | 1, 59 | 3.46 | .068 | 1, 71 | 0.41 | .524 |
|  | Day | 5, 295 | 1.64 | .148 | 5, 355 | 1.26 | .283 |
|  | Condition x Day | 5, 295 | 0.47 | .800 | 5, 355 | 0.61 | .690 |
| Sleep Duration | Condition | 1, 59 | 2.21 | .143 | 1, 71 | 0.45 | .505 |
|  | Day | 5, 295 | 4.64 | <.001 | 5, 355 | 0.98 | .427 |
|  | Condition x Day | 5, 295 | 0.31 | .905 | 5, 355 | 1.59 | .163 |
| *Note*. MVPA = moderate to vigorous physical activity (relative to the remaining time of the day). *N_Study1_* = 94; *N_Study2_* = 75. | | | | | | | |
|  | | | | | | | |
